# The instant impact of a single hemodialysis session on brain morphological measurements in patients with end-stage renal disease

**DOI:** 10.3389/fnhum.2022.967214

**Published:** 2022-08-23

**Authors:** Cong Peng, Qian Ran, Cheng Xuan Liu, Ling Zhang, Hua Yang

**Affiliations:** ^1^Department of Radiology, Chongqing Hospital of Traditional Chinese Medicine, Chongqing, China; ^2^Department of Radiology, Xinqiao Hospital, Chongqing, China; ^3^Laboratory for Cognitive Neurology, KU Leuven, Leuven, Belgium; ^4^Department of Nephrology, Chongqing Hospital of Traditional Chinese Medicine, Chongqing, China

**Keywords:** ESRD, hemodialysis, MRI, CAT12, cortical thickness

## Abstract

**Objective:**

To investigate the instant impact of hemodialysis (HD) on the cerebral morphological measurements of patients with end-stage renal disease (ESRD).

**Materials and methods:**

Twenty-five patients undergoing maintenance HD and twenty-eight age-, sex-, and education-matched healthy control (HC) were included. The HD group and HC group had 3D high-resolution structural magnetic resonance imaging (MRI) scans twice and once, respectively. Both groups underwent neuropsychologic tests. The morphological measurements of structural MRI were measured using CAT12 and these measures were compared among three groups. The relationship between morphological measures and clinical parameters and neuropsychological tests were investigated through multiple regression analysis.

**Results:**

Compared to the HC group, the cortical thickness before HD significantly decreased in the bilateral temporal lobe and significantly decreased in the left superior temporal gyrus after HD. The cortical thickness significantly increased in the bilateral temporal lobe, frontal lobe and occipital lobe after HD compared to before HD. The sulcus depth in the bilateral insula, frontal lobe, and parietal lobe after HD significantly increased compared to before HD. No significant differences in sulcus depth between HD and HC were detected. After HD, the cortical thickness of the right parsopercularis was positively correlated with the number connection test-A. Cortical thickness in multiple regions were positively correlated with blood flow velocity and cortical thickness in the left parahippocampal gyrus was negatively correlated with ultrafiltration volume. Patients showed better performance in the digit symbol test and line tracing test after HD compared to before HD, but there were no significant differences in the comparison of neuropsychologic tests between patients and HC.

**Conclusion:**

The instant morphological changes were captured during a single hemodialysis in HD patients. There was an association between these instant changes in the brain and clinical parameters and neuropsychologic tests. This work implied the instant impact of a single hemodialysis impact on the brain in HD patients.

## Introduction

End-stage renal disease (ESRD) is the final stage of chronic kidney failure and a leading contributor to morbidity and mortality worldwide (Liyanage et al., 2013). Morbidity associated with ESRD will probably enter a fast-growing period in the next few decades, associated with high morbidity of hypertension, diabetes, and other chronic diseases, especially in developing countries (Lozano et al., 2012; Liyanage et al., 2013; Coresh and Jafar, 2015; Wetmore and Collins, 2015, 2016). The treatment of this disease mainly relies on renal replacement therapy (RRT), including renal transplantation and dialysis. The second therapy is the mainstream for most ESRD patients. It has two types of strategy peritoneal dialysis and hemodialysis (HD). Most ESRD patients receive maintenance HD (Wetmore and Collins, 2015).

Accumulated evidence demonstrates that ESRD could cause cognitive impairment (Kurella et al., 2004). Cognitive impairment not only be related to the patient’s quality of life but also links to the patient’s treatment compliance and even higher mortality (Kurella et al., 2004; Madan et al., 2007; Hermann et al., 2014). Furthermore, ESRD patients who underwent maintenance HD had an even higher incidence of cognitive impairment (Fazekas et al., 1995; Ashwini et al., 1997; Murray et al., 2006; Bugnicourt et al., 2013; Chen et al., 2015).

The occurrence of cognitive impairment in ESRD might be due to renal failure, which causes the accumulation of neurotoxins in the body (Dong et al., 2018). These toxins could not be cleared in a timely and complete manner and the accumulated neurotoxins could lead to the nervous system damage and cognitive impairment (Bugnicourt et al., 2011; Popkov et al., 2019). Another reason for cognitive impairment might be associated with altered brain function and structure (Chou et al., 2013; Liang et al., 2013; Zhang et al., 2013, 2015a; Ni et al., 2014; Zheng et al., 2014; Chen et al., 2015; Ding et al., 2018). A lower value of region homogeneity (ReHo) was found mainly in the default mode network (DMN) in ESRD patients with HD (Chen et al., 2015) and multiple brain regions in ESRD patients with minimal nephrotic encephalopathy (MNE) (Liang et al., 2013). A correlation between their cognitive impairment and ReHo value was observed. Additionally, the functional connectivity (FC) among regions in which the ReHo decreased increased in HD patients compared to non-HD patients (Chen et al., 2015). Furthermore, the decreased FC in the DMN regions in ESRD patients with MNE was reported as well (Ni et al., 2014). The decreased FC of the frontal lobe was associated with cognitive impairment, anxiety, and decreased hematocrit (Zheng et al., 2014). Besides the changed FC of the brain in ESRD patients, the abnormal brain structure was reported as well. For instance, the white matter alterations were detected in ESRD through a diffusion tensor analysis. The axial diffusivity (AD), radial diffusivity (RD), and mean diffusivity (MD) values of the corpus callosum, bilateral sagittal stratum, and pons increased, but the fractional anisotropy (FA) value decreased (Chou et al., 2013). The FA values of the right anterior corona radiata and the left anterior thalamic radiation were significantly positively correlated with Mini Mental State Examination scores, but FA values of the bilateral superior longitudinal fasciculus were significantly negatively correlated with the TMT-B time consumption (Zhang et al., 2015a). The gray matter alterations were also detected in ESRD patients. For example, the gray matter volume decreased and it was associated with cognitive impairment. The serum urea level may be a factor to affect the gray matter volume (Zhang et al., 2013). A decreased gray matter volume and FC patterns of the putamen were observed in ESRD patients with sensorimotor abnormalities (Ding et al., 2018).

To our knowledge, most studies mainly focused on the cross-sectional comparative observation between ESRD patients and HC or between different dialysis patients and HC (Jiang et al., 2016; Luo et al., 2016; Cheng et al., 2019). Few studies had worked on the impact of a single HD process on the brain in ESRD so far (Li et al., 2018). Therefore, this study investigated the instant impact of a single HD process on brain morphological changes that potentially uncover the mechanism of cognitive impairment. Meanwhile, the study compared the changes in brain structure between patients and HC to observe the impact of periodic HD sessions on brain structure. Through observing the possible relationship between instant and basic status change, we explored whether a single HD affected the basic status of brain structure.

## Materials and methods

All ESRD underwent maintain HD patients were included in Chongqing Hospital of Traditional Chinese Medicine, Chongqing, China. A total of 36 patients (20 males; mean age 56.22 ± 11.76 years; range 27–76 years) and 29 age- (mean age 51.07 ± 11.36 years; range 29–68 years), sex- (11 males), and education-matched volunteers were included. The exclusion criteria were the same for both groups as follows: (1) brain lesions, such as severe craniocerebral trauma, stroke, and brain tumor; (2) drug or alcohol abuse; (3) neurological diseases, such as Alzheimer’s disease, Parkinson’s disease; and (4) psychiatric issues. This study was approved by the local ethics committee and has been performed in accordance with the ethical standards laid down in the 1964 Declaration of Helsinki and its later amendments. Written informed consent for all participants was obtained. All patients received magnetic resonance imaging (MRI) scans and neuropsychological tests twice, around 1 h before HD (HDb) and within half an hour after the HD (HDa). The observation time point was set to be as close as possible to the HD process to reflect the instant impact of the HD process on brain structure. All HC subjects underwent MRI scan and neuropsychological tests once.

### Imaging acquisition

Magnetic resonance imaging data were acquired using a 1.5-T MR scanner (Avanto; Siemens Medical Solutions, Erlangen, Germany) with an 8-channel phased-array head coil. The 3D high-resolution T1-weighted images were obtained through the three-dimensional magnetization-prepared rapid gradient-echo (3D-MPRAGE) sequences: TE = 3.25 ms, TR = 2,200 ms, image matrix, FOV = 256 mm × 192 mm, slice thickness = 1 mm, flip angle = 8°, number of slices = 176. Foam padding was fitted to reduce head motion. Subjects were instructed to try and remain motionless throughout the acquisition of the images.

### Image quality checking

In order to ensure that there were no apparent structural abnormalities or artifacts, image quality was evaluated by an experienced radiologist (HY) with 15 years of experience in neuroradiology who was blinded to whether the images were from the HD group or HC group. Besides, CAT12 was used to evaluate the image quality as well. Based on the Cat report generated from CAT12, image quality was divided into Grades A-D. Subjects with grade C or worse or the extracted cortex area which was mismatched to the non-cortical area were excluded from this study (Schmitgen et al., 2019). A total of 11 out of 36 HD subjects were excluded due to brain lesions (3 subjects) or quitted the second scan (two subjects) or image quality rated C or worse (six subjects), and 1 out of 29 HC subjects was excluded due to image quality rated C.

### Structure magnetic resonance imaging preprocessing

The vertex-wise surface-based morphometry (SBM) method was used to analyze the brain structure. We conducted it through Computational Anatomy Toolbox (CAT12)^1^ (a toolbox attached to software package SPM 12).^2^ CAT12 is an efficient brain segmentation tool (Fellhauer et al., 2015; Tavares et al., 2020; Fu et al., 2021). It has a relatively small amount of calculation with a fast calculation speed and it can measure the brain surface and brain complexity.

Based on the projection-based thickness (PBT) method (Dahnke et al., 2013), CAT12 automatically estimated the cortical thickness of the brain. All images were automatically segmented into gray matter (GM), white matter (WM), and cerebrospinal fluid (CSF). We then applied warping of segmented images to the Montreal Neurological Institute (MNI) space using the deformation field obtained from the segmentation step. This workflow included topology correction, spherical mapping and registration, and partial volume estimation. The cortical thickness, sulcus depth, and the whole brain indices including volume of CSF, GM, and WM were extracted. Then, the data were resampled and smoothed.

### Neuropsychological tests

As ESRD patients receiving dialysis exhibit memory disturbance, impaired attention, slow motor performance, depression, and anxiety (Luo et al., 2016), we used the battery of neuropsychological tests in the following to assess ESRD patients’ cognitive impairment. The battery of neuropsychological tests (Ferenci et al., 2002; Bajaj et al., 2009; Liang et al., 2013; Zhang et al., 2013, 2015b; Jiang et al., 2016) included the digital symbol test (DST), number connection test-A (NCT-A), line tracing test (LTT), serial dots test (SDT), the Hamilton Anxiety Scale (HAMA) and Hamilton Depression Scale (HAMD) (Lin et al., 2018). The DST mainly assesses the subject’s attention, visual memory ability, and psychomotor speed. The NCT-A mainly assesses psychomotor speed. The LTT mainly assesses speed and accuracy. The SDT mainly assesses attention, visual memory ability, and psychomotor speed. The HAMA and HAMD were used to evaluate anxiety and depression, respectively. To reduce the interference of the learning effect, all the subjects were trained before tests.

### Statistical analysis

Demographic and neuropsychological tests were performed using SPSS 24 (IBM, Armonk, NY, United States). An independent two-sample *t*-test was used to investigate differences in age and education between the HD and HC groups. The chi-square test was used to compare sex differences. The differences in performance on the neuropsychological tests were compared using the independent two-sample *t*-test. The paired *t*-test was employed to compare performance on the neuropsychological tests, total GM volume, WM volume, and CSF volume between the HDb and HDa groups.

Cortical thickness and sulcus depth of HDb and HDa were compared with the HC group, respectively, using the independent two-sample *t*-test (sex, age, and education as covariates). Cortical thickness and sulcus depth between HDb and HDa were compared using the paired *t*-test.

We used multiple regression in SPM12 to evaluate: (1) the associations between cortical thickness and sulcus depth in two groups (HDb and HDa) and age, education, sex, duration of dialysis, duration of disease; (2) the associations between cortical thickness and sulcus depth of HDa and ultrafiltration volume, blood flow velocity, and performance on neuropsychological tests. For all vertex-based analyses, the threshold was set at *p* < 0.05 with FWE-corrected.

## Results

### Demographic and neuropsychological tests

Demographic and neuropsychological tests for all subjects were listed in Table 1. There were no significant differences in sex (*p* = 0.353), age (*p* = 0.168), or education (*p* = 0.841) between the HD and HC groups. There were no significant differences in performance on neuropsychological tests between the HDb group and HC group (NCT-A: *p* = 0.64, DST: *p* = 0.07, LTT *p* = 0.91, SDT *p* = 0.41) while the mean score of neuropsychological tests of the HDb group was lower than that of the HC group. There were no significant differences in performance on neuropsychological tests between the HDa group and HC group either (NCT-A: *p* = 0.90, DST: *p* = 0.32, LTT *p* = 0.34, SDT *p* = 0.99). The DST score of the HDa group was significantly higher than that of HDb group (*p* = 0.013), and the LTT consume time of HDa group was significantly shorter than that of HDb group (*p* = 0.016). There were no significant differences in performance on the NCT-A and SDT tests between the HDa group and HDb group (*p* = 0.256, 0.138).

**TABLE 1:** Demographic and clinical measures.

Parameter	HD (*n* = 25)		HC (n = 28)	*P-value*		
	HDb	HDa				
				HC vs. HDb	HC vs. HDa	HDa vs. HDb
Sex (M/F)	13/12		11/17	0.35	0.35	
Age (years)	55.28 ± 13.04		50.61 ± 11.29	0.17	0.17	
Education (years)	12.24 ± 3.19		12.04 ± 4.09	0.84	0.84	
NCT-A (sec)	70.13 ± 28.92	64.19 ± 34.22	65.54 ± 39.91	0.64	0.90	0.27
DST	37.38 ± 15.52	40.62 ± 16.08	46.07 ± 20.79	0.07	0.32	0.01
LTT (sec)	72.29 ± 25.62	64.14 ± 26.38	71.11 ± 24.07	0.91	0.34	0.02
SDT (sec)	52.00 ± 12.60	48.90 ± 14.35	48.89 ± 14.79	0.41	0.99	0.14
HAMA	8.63 ± 4.29		8.07 ± 5.66	0.70		
HAMD	10.04 ± 5.81		7.93 ± 6.08	0.21		

Data are expressed as the means ± standard deviation. M, male; F, female; sec, second.

One patient failed all neuropsychological tests, and four patients failed the NCT-A and LTT in the HDa group.

### Changes in volume of gray matter, white matter, and cerebrospinal fluid

Compared to the HDb group, the volume of CSF in the HDa group was significantly decreased, but the volume of GM and WM was significantly increased (*p* < 0.05; Table 2).

**TABLE 2:** Comparison of global volume of GM, WM, and CSF before and after dialysis.

	Paired differences			
	Mean value	SD	*t*-Value	*P-value*
CSF-vol: HDa-HDb	−42.36	53.89	−3.93	0.001
GM-vol: HDa-HDb	28.44	19.07	7.45	<0.001
WM-vol: HDa-HDb	16.84	7.21	11.68	<0.001
CSF-CR: HDa-HDb	−3.06	2.58	−5.92	<0.001
GM-CR: HDa-HDb	1.93	1.31	7.40	<0.001
WM-CR: HDa-HDb	1.11	1.55	3.58	0.002

CSF, cerebrospinal fluid; GM, gray matter; WM, white matter; vol, volume; CR, constituent ratio; paired differences: paired difference of cortical thickness between HDb and HDa.

### Changes in cortical thickness

**FIGURE 1:**
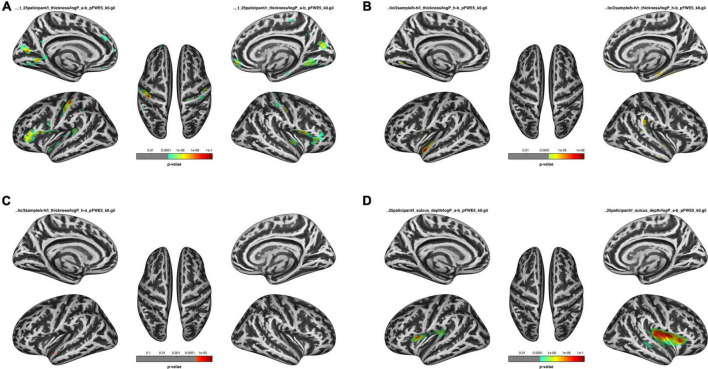
Comparison of before HD (HDb), after HD (HDa), and healthy control (HC) groups. **(A)** Comparison of the thickness of the HDa group with that of the HDb group. **(B)** Comparison of the thickness of the HDb group with that of the HC group. **(C)** Comparison of the thickness of the HDa group with that of the HC group. **(D)** Comparison of the sulcus of the HDa group with that of the HDb group.

Comparisons of cortical thickness among the HDb, HDa, and HC groups were listed in Figure 1 and Table 3.

**TABLE 3:** Regions showing cortical thickness differences among the before HD (HDb), after HD (HDa), and healthy control (HC) groups.

	Side		Overlap of atlas region		
		Number of vertexes	*P-value*	Region	Proportion
**Thickness**					
HDa > HDb	L	3,031	<0.001	Parstriangularis	47%
				Parsopercularis	28%
				Precentral	21%
	L	1,613	<0.001	Postcentral	97%
	L	725	<0.001	Pericalcarine	53%
	L	703	<0.001	Lingual	72%
	L	672	<0.001	Superiortemporal	100%
	R	2,136	<0.001	Parstriangularis	48%
				Parsopercularis	32%
	R	921	<0.001	Lingual	100%
	R	602	<0.001	Superiortemporal	56%
				Insula	44%
	R	600	<0.001	Cuneus	75%
				Precuneus	21%
	R	397	<0.001	Postcentral	100%
HC > HDb	L	824	<0.001	Superiortemporal	98%
	L	125	<0.001	Precentral	100%
	L	77	<0.001	Lingual	100%
	R	343	<0.001	Fusiform	100%
	R	319	<0.001	Supramarginal	100%
	R	191	<0.001	Parahippocampal	93%
	R	145	<0.001	Superiortemporal	100%
HC > HDa	L	103	<0.001	Superiortemporal	100%

The paired sample *t*-test was used to compare differences between the HDb and HDa, and the independent two-sample *t*-test was used to compare differences between the HDb and HC groups and the HDa and HC groups; thickness comparison *p* < 0.05, FWE correction.

Compared to the HC group, the HDb group showed significantly thinner thickness in the left superior temporal gyrus, precentral gyrus, right fusiform gyrus, supramarginal gyrus, parahippocampal gyrus, and superior temporal gyrus (*p* < 0.05, FWE corrected).

Compared with the HC group, the HDa group showed significantly thinner thickness in the left superior temporal gyrus (*p* < 0.05, FWE corrected).

Compared with the HDb group, the HDa group showed increased thickness in the left parstriangularis, parsopercularis, precentral gyrus, postcentral gyrus, pericalcarine, cuneus, lingual gyrus, superior temporal gyrus, right parstriangularis, parsopercularis, lingual gyrus, superior temporal gyrus, insula, cuneus, precuneus, and postcentral gyrus (*p* < 0.05, FWE corrected).

### Changes in sulcus depth

There were no significant differences in sulcus depth between the HDb and HC groups or between the HDa and HC groups.

However, compared to the HDb group, the HDa group showed increased sulcus depth in the left insula, supramarginal gyrus, parsopercularis, precentral gyrus, postcentral gyrus, transverse temporal gyrus, right insula, postcentral gyrus, superior temporal gyrus, transverse temporal gyrus, supramarginal gyrus, parsopercularis, parstriangularis, and precentral gyrus (*p* < 0.05, FWE correction at the cluster level, initial vertex-wise threshold *p* = 0.001; Figure 1 and Table 4).

**TABLE 4:** Regions showing cortical sulcus depth differences among the before HD (HDb), after HD (HDa), and healthy control (HC) groups.

	Side		Overlap of atlas region		
		Number of vertexes	*P-value*	Region	Proportion
**Sulcus depth**					
HDa > HDb	L	3,276	<0.001	Insula	35%
				Supramarginal	15%
				Parsopercularis	12%
				Postcentral	10%
				Precentral	10%
				Transversetemporal	9%
				Superiortemporal	6%
	R	8,404	<0.001	Insula	40%
				Postcentral	11%
				Superiortemporal	9%
				Transversetemporal	9%
				Supramarginal	9%
				Parsopercularis	8%
				Precentral	8%
				Parstriangularis	6%

The paired sample *t*-test was used to compare differences between the HDb and HDa groups; sulcus depth comparison *p* < 0.05, FWE correction at the cluster level, initial vertex-wise threshold *p* = 0.001.

### The associations of cortical thickness with clinical measures and neuropsychological tests

**FIGURE 2:**
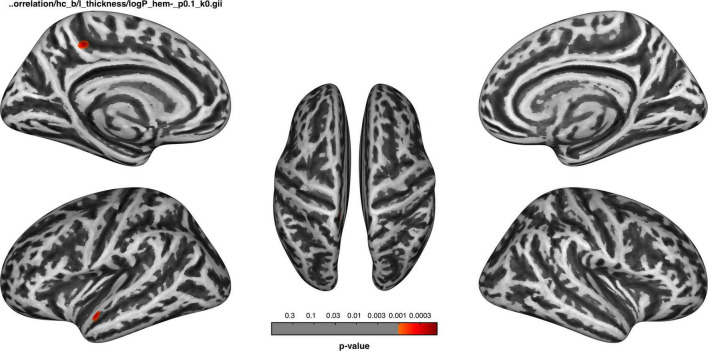
Before dialysis, the thickness of the left superior temporal cortex and precuneus was negatively correlated with the duration of dialysis.

**FIGURE 3:**
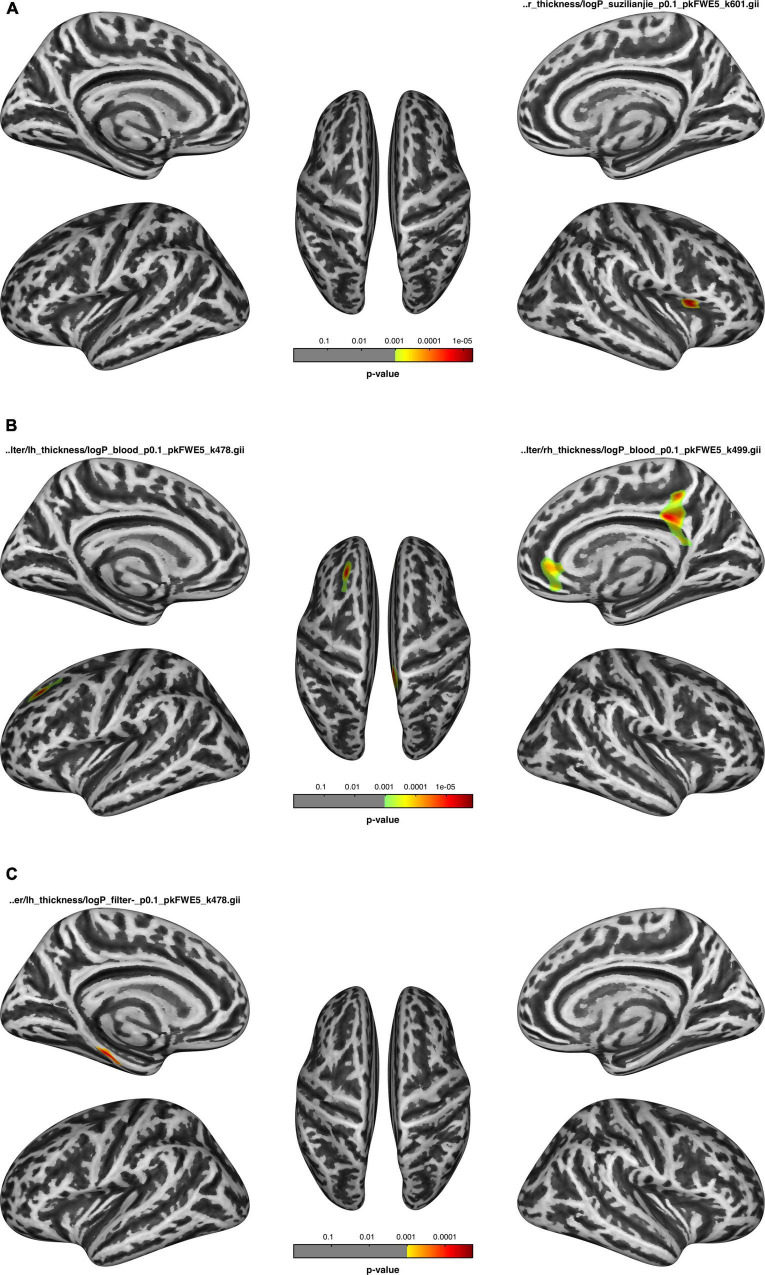
After dialysis, **(A)** the thickness of the regions correlated with the number connection test-A (NCT-A); **(B)** the thickness of the regions correlated with blood flow velocity; **(C)** the thickness of the regions negatively correlated with ultrafiltration volume.

Associations between cortical thickness and clinical measures and neuropsychological test analyses are shown in Figures 2, 3 and Table 5.

**TABLE 5:** Correlation between cortical thickness of after HD (HDa) and number connection test-A (NCT-A), Blood flow velocity, ultrafiltration volume.

	Side		Overlap of atlas region		
		Number of vertexes	*P-value*	Region	Proportion
**Positive correlation**					
NCT-A	R	462	<0.001	Parsopercularis	87%
Blood flow velocity	L	463	<0.001	Superiorfrontal	94%
	R	1,534	<0.001	Isthmuscingulate	52%
				Precuneus	30%
				Posteriorcingulate	16%
	R	544	<0.001	Rostralanteriorcingulate	61%
				Medialorbitofrontal	39%
**Negative correlation**					
Ultrafiltration volume	L	409	<0.001	Parahippocampal	100%

Multiple regression for correlation analysis, covariates: age, sex, education, duration of dialysis, duration of disease.

The cortical thickness of the left superior temporal and precuneus of the HDb group was negatively correlated with the duration of dialysis, the significance level was set at an uncorrected *p* < 0.001, with a cluster size > 50 vertices, but not survived after FWE correction (Figure 2).

The cortical thickness of the right parsopercularis of the HDa group was positively correlated with the NCT-A (Figure 3A); the thickness of the left superior frontal, right isthmus of the cingulate, precuneus, posterior cingulate, rostral anterior cingulate, and medial orbitofrontal gyrus was positively correlated with blood flow velocity (Figure 3B); and the thickness of the parahippocampal gyrus was negatively correlated with ultrafiltration volume (Figure 3C; *p* < 0.05, FWE correction at the cluster level, initial vertex-wise threshold *p* = 0.001).

## Discussion

This study focused on the instant impact of a single HD on the brain structure in ERSD. The results of our study demonstrated changes in brain morphology during a single HD were observed in ESRD patients, the cortical thickness change in left superior temporal gyrus was captured in all the comparison among three groups. The instant changes in the cortical thickness were associated with clinical parameters and neuropsychologic tests. Patients showed better performance in the digit symbol test and line tracing test after HD compared to before HD.

We demonstrated that the volumes in GM and WM increased but decreased in CSF after a single HD. This might be caused by significant changes in blood volume (Polinder-bos et al., 2018), especially blood osmolality (Schmitgen et al., 2019) due to the HD process. Silver et al. (1992) found that the plasma urea of rats decreased from 72 to 34 mmol/L, plasma osmolality decreased 8% (29 mOsm/kg), and brain water increased 6% with 90 mins HD. During the HD process, a large number of small molecules were removed, resulting in a dramatic change in osmolality on both sides of the blood-brain barrier (Silver et al., 1996; Boulard, 2001). The water molecules in the blood entered the interstitium as plasma osmolality decreases, leading to an increase in gray matter volume and white matter volume, and decreasing the CSF volume by compression (Silver et al., 1996; Boulard, 2001). The performance on neuropsychological tests showed that subjects performed better after the HD process. Indicated that the increase in brain volume did not cause instant cognitive impairment in HD patients. Therefore, we thought that the change in osmolality caused by HD did not lead to excessive swelling of the brain. There might be some positive factors that antagonize the volume change caused by osmolality.

One of these positive factors might be the recovery of cell function, followed by the clearance of uremic toxins. Popkov et al. (2019) reported that mitochondria were not only the target of uremic toxins but also the important organelle of uremic toxin production. The clearance of uremic toxins could promote the recovery of mitochondrial function, providing energy for cell activities (McBride et al., 2006; Popkov et al., 2019). It would benefit the metabolism of cells and the stability of the internal environment. Additionally, more energy could help maintain cell electrophysiology and accelerate the recovery of synaptic signal transmission (Matsuda et al., 1968; McBride et al., 2006), which might benefit cognition. In this work, although the cortical thickness increased after HD, there were no regions thicker than that of the HC group. Therefore, it could be confirmed that the increase in cortical thickness was positively limited and it didn’t cause brain edema.

The cortical thickness of the bilateral parstriangularis, parsupercularis, lingual gyrus, superior temporal gyrus, posterior central gyrus, cuneus, right precuneus, insula, left central gyrus, and pericalcarine increased after a single HD compared to the HDb group. The regions in which their sulcus depth increased after a single HD partially overlapped with or adjacent to the regions in which their cortical thickness altered compared to the HDb group. This might indicate that brain regions have different sensitivities to severe changes in cerebral hemodynamics and the internal environment during HD. This was consistent with the findings in previous studies (Venkateshappa et al., 2012). Song et al. (2008) using F-18-fluorodeoxyglucose positron emission tomography demonstrated that the metabolism of multiple brain regions in patients with chronic kidney disease but without HD was lower than that in HC. Some of these brain regions (e.g., bilateral temporal lobe, frontal lobe) with decreased cerebral glucose metabolism were similar to cortical thickness changed regions after HD in this study, which may indicate that these brain regions might have more metabolic changes than others. Cerebral hemodynamics changes were found in dialysis patients using arterial spin labeling MRI. Compared to non-dialysis patients, regional cerebral blood flow of dialysis patients decreased mainly in bilateral frontal and anterior cingulate cortices (Jiang et al., 2016), which was partially overlapped with regions changed thickness in this study. This may indicated that the decrease in blood perfusion may participate in cortical thickness changes. Li et al. (2019) reported that brain sulcal depth, gyrification, and cortical thickness changes occurred in chronic users of codeine-containing cough syrups. We found that changes in the bilateral frontal, temporal, and occipital lobes were coincident with those in this study. It may indicate that these regions were vulnerable by stimulation.

Patients in HDa group performed better in the DST and LTT than HDb group, but presented a similar performance as HC group in all neuropsychological tests. We hypothesized that the HD process did not induce an instant negative impact on the cognition of the patients and even might have improved it.

There were no significant differences in the HAMA score and HAMD score between the HD and HC groups. As studies showed that the incidence of anxiety and depression in ESRD patients was higher than that in the general population, anxiety and depression could affect patients’ cognition (Palmer et al., 2013; Kielstein et al., 2015; Spoto and Zoccali, 2015). Therefore, there were no effects of anxiety and depression on instant changes in brain morphology.

Interestingly, in the comparison among the three groups, we found that the cortical thickness of the left superior temporal gyrus decreased in both HD groups compared to that of HC group. However, the cortical thickness of this region increased within a single HD process. Furthermore, the thickness of the left superior temporal gyrus was negatively correlated with the duration of dialysis. Although the result was uncorrected (no vertex survived with FWE correction), it was just in the left superior temporal gyrus coincidentally. Therefore, we concluded that the changes in basic status may be accumulated by periodical changes of single HD session. Polinder-bos et al. (2018) reported changes in blood volume during a single HD and thought that a single periodic change in blood volume may accumulate to cause brain injury.

The superior temporal gyrus participated in the process of auditory and linguistic information, it was related to social cognition (Bigler et al., 2007; Dziobek et al., 2011). According to the research of Montoliu et al. (2012), the change in cortical thickness of the superior temporal gyrus was involved in the cognitive impairment of mild hepatic encephalopathy.

The cortical thickness of the right parsopercularis was positively correlated with the NCT-A indicating that the changes in cortical thickness had an impact on patients’ cognition.

The changes in cortical thickness were associated with ultrafiltration volume and blood flow velocity, which were important parameters that could be set and adjusted to the patient’s condition in the HD process (Daryani et al., 2020). It was suggested that these two parameters need to be seriously considered when planning HD treatment. We should not blindly pursue a more thorough and faster HD process.

Our study had several limitations. First, the understanding of the specific mechanism of cortical thickness changes and cognitive improvement in a short period was based on the speculation of data analysis, which lacks the support of laboratory research and needs further study. Second, this study attempted to observe the instant impact of the HD process on the brain, but the HD process was a dynamic process. Limited by current technology, it was impossible to monitor the changes in the brain in real-time during the HD session. The development of technology may help to realize real-time monitoring in the process to better reveal the mechanism of the impact of the HD process on the brain. Third, the sample size was relatively small.

In summary, the changes in brain morphology during a single HD were observed in ESRD patients. These changes showed a significant association with clinical parameters and neuropsychological tests. This instant impact of HD might be accumulated in the left superior temporal gyrus.

## Data availability statement

The original contributions presented in this study are included in the article/supplementary material, further inquiries can be directed to the corresponding author.

## Ethics statement

The studies involving human participants were reviewed and approved by Ethics Committee of Chongqing Hospital of Traditional Chinese Medicine. The patients/participants provided their written informed consent to participate in this study.

## Author contributions

HY contributed to the conception of the study. CP, CL, and LZ performed the experiment. QR and CP contributed significantly to analysis, performed the data analyses, and wrote the manuscript. All authors contributed to the article and approved the submitted version.

## Footnotes

1
http://dbm.neuro.uni-jena.de/cat/

2
http://www.fil.ion.ucl.ac.uk/spm/software/spm12
